# Time trends analysis of statin prescription prevalence, therapy initiation, dose intensity, and utilization from the hospital information system of Jinshan Hospital, Shanghai (2012–2018)

**DOI:** 10.1186/s12872-020-01482-5

**Published:** 2020-04-25

**Authors:** Yujuan Liu, Xiaoqun Lv, Ning Xie, Zhonghong Fang, Weifang Ren, Yuan Gong, Yan Jin, Jun Zhang

**Affiliations:** 1 Department of Clinical Pharmacy, Jinshan Hospital Affiliated to Fudan University, Shanghai, 201508 China; 2 Department of Clinical Pharmacy, Zhongshan Hospital Qingpu Branch Affiliated to Fudan University, Shanghai, 201799 China; 3 Shihua Community Health Service Center, Jinshan District, Shanghai, 200540 China

**Keywords:** Statins, Prevalence, Cardiovascular disease, Initiation, Preventative intervention

## Abstract

**Background:**

Statin remains a mainstay in the prevention and treatment of cardiovascular diseases. Statin utilization has evolved over time in many countries, but data on this topic from China are quite limited. This study aimed to investigate the changing trends of statins prescription, as well as detail the statin utilization through a successive longitudinal study.

**Methods:**

The prescription database was established based on electronic health records retrieved from the hospital information system of Jinshan Hospital, Fudan University from January 2012 to December 2018 in Shanghai, China. The prescription rates and proportions of different statin types and doses among all patients were examined. Sub-analyses were performed when stratifying the patients by age, gender, dose intensity, and preventative intervention.

**Results:**

During the study period, a total of 51,083 patients, who were prescribed for statins, were included in this study (mean [SD] age, 59.78 [±13.16] years; 53.60% male, *n* = 27, 378). The overall statins prescription rate in which patients increased from 2012 (1.24, 95% CI: 1.21-1.27%) to 2018 (3.16, 95% CI: 3.11–3.20%), *P* < 0.001. Over 90% of patients were given a moderate dose of statins. Patients with a history of coronary and cerebrovascular events (over 32%) were more likely to be prescribed with statins for preventative intervention. Furthermore, our study has witnessed a significant rise in statin therapy in primary and secondary prevention.

**Conclusions:**

In conclusion, statins were frequently prescribed and steadily increased over time in our study period. There were also changes in statin drug choices and dosages. A coordinated effort among the patient, clinical pharmacist, stakeholders and health system is still needed to improve statin utilization in clinical practice in the future.

## Background

The incidence of cardiovascular diseases (CVD) continues to rise and has become the leading cause of mortality (responsible for above 40% of all deaths) in China in recent years [1]. Statins, the professional name of 3-hydroxy-3-methylglutaryl coenzyme A reductase inhibitors, have been proven to lower the morbidity and mortality of cardiovascular events and widely used in prevention in patients with CVD [2]. It is recommended as the most effective lipid-lowering drug at present, which can not only effectively reduce total cholesterol (TC) but also low-density lipoprotein (LDL) [3–5].

Cholesterol plays a crucial role in the pathogenesis of coronary heart disease (CHD) and Atherosclerotic cardiovascular disease (ASCVD), and it has been a global consensus to prevent and control the cardiovascular risk of ASCVD by reducing blood LDL cholesterol (LDL-C) level [6]. The American College of Cardiology (ACC)/American Heart Association (AHA) 2013 guidelines (2013 ACC/AHA) cholesterol guidelines recommend that all patients with ASCVD should receive high-dose or moderate-dose statins therapy while ignoring lipid targets, and have recommended statin therapy to a specific group for primary and secondary prevention [7]. The 2016 European Society of Cardiology (ESC)/the European Atherosclerosis Society (EAS) (2016 ESC/EAC), the most widely used lipid management guideline, still targets lipid levels at different stages of disease activity before recommending statins [8]. Based on the 2007 Chinese Guidelines for the Management of Dyslipidemia in Adults, the 2016 Chinese guideline for the management of dyslipidemia in adults (referred to as “the new Guideline” hereafter) was released by Chinese Journal of Cardiology in 2016 formulated by a joint committee of multidisciplinary experts. It is not only in line with other important international guidelines but also has its own recommendations. It emphasizes the critical role of cholesterol on ASCVD. The new guidelines highlight the overall cardiovascular risk assessment, the use of LDL-C as the preferred intervention target (Class I recommendation, Level A evidence) and some other aspects (for more details, please refer to [9]). The introduction and popularization of the new guidelines will greatly increase the confidence of clinicians in statins utilization and contribute to more standardized use of statins in China.

Statins rank the most commonly prescribed medications in many countries, and general increase trends have been witnessed worldwide. In the United States (US), statin users in adults who reported using any statin observed a 79.8% increase from 17.9% (2002-2003) to 27.8% (2012-2013) [10]. They also reported a steady increase among patients without ASCVD, those with diabetes and those with hyperlipidemia and not diabetes over the 12 years. From another study in the UK, prescription prevalence increased sharply from 1995 to 2013. Meanwhile, statin therapy initiation rates rose sharply from 1995 to 2006 [11]. In addition, the proportion of high-intensity statin increased from 16.5% in (2002–2003) to 20.4% (2012–2013) in the general adult population [10]. Similarly, prescription of high-intensity statins significantly increased, particularly, among patients with cerebrovascular accidents (CVA) [12] and coronary artery disease [2]. By contrast, high-intensity statins use remained low in Taiwan [13] and Hong Kong [14]. Although the 2013 ACC/AHA guideline recommends the initiation of high-intensity statin therapy in patients with ASCVD regardless of baseline low-density lipoprotein (LDL) cholesterol levels.

Over the past two decades, accumulating evidence has shown the real benefits of different statins in reducing the risk of cardiovascular events (including myocardial infarction, coronary heart death, and ischemic stroke). In addition to that, several large-scale clinical trials have consistently proven that statins could play a significant role in both primary and secondary prevention. These studies also expanded the scope of statin application from patients with ASCVD to primary prevention group and even more extensive populations. Recent studies indicated that cumulative exposure to lipids plays a critical causative role in the initiation and progression of atherosclerosis [15]. Patients who tend to be at risk for developing atherosclerosis will benefit most from statin therapy by the maintenance of the optimal lipid levels from early in life [16]. However, the clinical effect in the population at low risk of cardiovascular disease still needs further study.

Also, statins have been the most commonly prescribed drugs in China in recent decades. Currently, plenty of medicaments of statins have entered the market. Frequently used statins in China include lovastatin, simvastatin, pravastatin, fluvastatin, atorvastatin, rosuvastatin, and pitavastatin. A previous study has shown that the overall statins prescription rate in patients with a discharge diagnosis with ASCVD was 58.8% in West China Hospital from 2008 to 2014. It has an average 10–20% lower rate when compared with that in western or developed countries [17]. Except for a few studies [11, 13, 14], little is known about the detailed utilization of statins. Thus, in this study, we will not only investigate the change trends of statins but also demonstrate statin prescription prevalence, therapy initiation, dose intensity, and its utilization in preventative interventions in a tertiary hospital of China for a successive 7 years. The result of this comprehensive study will contribute to form the general clinical practice in statin use and provide evidence-based guidelines in China.

## Methods

### Data source and sample selection

This study is a consecutive cross-sectional study based on longitudinal monitoring data analysis focusing on patients receiving any statin treatment from January 1, 2012, to December 31, 2018. Data were retrieved from the hospital information system (HIS) of Jinshan hospital affiliated to Fudan University, which is the only third-tier hospital in the catchment area. It has 1000 regular hospital beds. In 2016, the hospital has handled 1.47 million outpatient and emergency visits. The related information was withdrawn from patients receiving their first statin therapy during the study period. The prescription contains the medical card number, patient name, gender, age, diagnosis, generic name of statin and dosage. Different types of statins including atorvastatin, simvastatin, rosuvastatin, pravastatin, fluvastatin prescription were analyzed. We obtained the registered population, gender and age composition of Jinshan district from 2012 to 2018 through the statistical yearbook of Jinshan District, Shanghai.

### Inclusion/exclusion criteria

Patients were deemed to be prescribed statins once when they received any types and dosages of statins. The exposure of statin-treated patients was manually examined by two reviewers. Only individuals aged 18 years or above were included in our study. Patients with a missing medical card number or an unknown date of birth or gender were excluded. We also excluded patients who changed their prescribed statins in a calendar year.

### Statin prescription rate

Statin prescription data were retrieved from all prescriptions containing any dispensation of atorvastatin, fluvastatin, pravastatin, rosuvastatin or simvastatin. A statin user was considered and defined if they had at least one statin prescription dispensing in a given calendar year. The dose, intensity, and frequency of each drug were retrieved and analyzed.

Statin prescription rate included two measurements: total and new prescription rate. For the total prescription rate of a specific statin agent, we calculated the number of patients prescribed with a specific statin agent, then divided by the total number of statin users in the year. Similarly, we calculated the new prescription rate by referring to the previous study [13].

### Statin therapy initiation rate and prescription prevalence

We defined initiation of statin therapy if a patient, who was not on a statin during a 365-day washout period (new statin users), was prescribed any statin prescription before admission. The statin therapy initiation rate was calculated as previously described as the total number of new statin users per year divide by the annual population at risk [14]. Furthermore, we also analyzed the statin initiation rate according to gender, age group, statin intensity, and preventative intervention.

The statin prescription prevalence was estimated by using the total number of patients with any statin prescription during each calendar year as the numerator, while the annual total population in the hospital catchment area as the denominator. Prevalence was also stratified according to gender, age group and statin dose intensity. All statin prescriptions in this study were from 2012 to 2018. We took 2012 as the baseline to study the prescription prevalence rate of new statin users from 2013 to 2018.

### Statin dose intensity

Statin dose intensity was defined as per the guidelines for the prevention and treatment of dyslipidemia in Chinese adults (2016) [18], ACC/AHA guideline on the treatment of blood cholesterol [7] and definition as previously described [13]. (1) low-intensity statins was defined as atorvastatin < 10 mg/day, rosuvastatin < 5 mg/day, simvastatin < 20 mg/day, pravastatin < 40 mg/day and fluvastatin < 80 mg/day; (2) moderate-intensity statins was defined as 10 mg/day ≦ atorvastatin < 40 mg/day, 5 mg/day ≦ rosuvastatin < 20 mg/day, 20 mg/day ≦ simvastatin < 80 mg/day, pravastatin ≧ 40 mg/day, and fluvastatin ≧80 mg/day; (3) high-intensity statins was defined as atorvastatin ≧40 mg/day, rosuvastatin ≧20 mg/day and simvastatin ≧80 mg/day.

### Primary and secondary prevention of cardiovascular disease

Primary and secondary prevention have a major role in the fight against cardiovascular diseases. We defined primary and secondary prevention based on criteria of the China Cholesterol Education Program (CCEP) in 2014 and the Chinese guidelines for the prevention and treatment of dyslipidemia in adults in 2007 [19]. We also referred ICD-10 (International Classification of Diseases) diagnosis codes [20].

### Statistical analysis

Statistical analysis was performed by the application of SAS (version 9.2). Baseline characteristics of patients were summarized using frequencies and proportions for categorical data. Continuous variables were expressed as mean ± standard deviation. 95% confidence intervals for the observed prevalence and initiation rates were calculated. Cochran-Armitage test for trend was applied to analyze overall estimates of statin prescription prevalence in consecutive years. Comparisons of the proportions were carried out using Pearson’s chi-squared test, and continuous variables were compared using analysis of variance (ANOVA) or Student’s *t*-test as appropriate. A *p*-value < 0.05 was considered statistically significant for all analyses.

## Results

### Patient demographics and statin prescription

Overall, the total number of patients exposing to statin between January 1, 2012, to December 31, 2018, was 51,083 with an average age of 59.78 years. The proportion of males reached up to 53.60% (*n* = 27, 378) of the overall population. The age band with a maximum number of patients was 35–59 years old, with a total of 23,006 patients accounting for 45.04%, followed by age group 65–79 (*n* = 14,842, accounting for 29.05%). Patient demographics and statin therapies are described in Table 1. We in total analyzed 279, 223 statin prescriptions during the study period. Rosuvastatin (*n* = 188,823, 67.62%) was the highest-ranked statin in investigated patients, followed by atorvastatin (*n* = 45,974, 16.47%) and simvastatin (*n* = 30,718, 11.00%).

**Table 1:** Patient demographics of statin users and statin prescriptions from the hospital information system of Jinshan Hospital, Shanghai

Characteristics	Value (%)
Gender	
Male	27,378 (53.60)
Age	
18–34 years	1679 (3.29)
35–59 years	23,006 (45.04)
60–64 years	7971 (15.60)
65–79 years	14,842 (29.05)
+ 80 years	3585 (7.02)
Average age	59.78
Total prescriptions	279,223
simvastatin	30,718 (11.00)
rosuvastatin	188,823 (67.62)
ravastatin	9624 (3.45)
atorvastatin	45,974 (16.47)
fluvastatin	4084 (1.46)

### Choice of statin and prescription rate

**Fig. 1:**
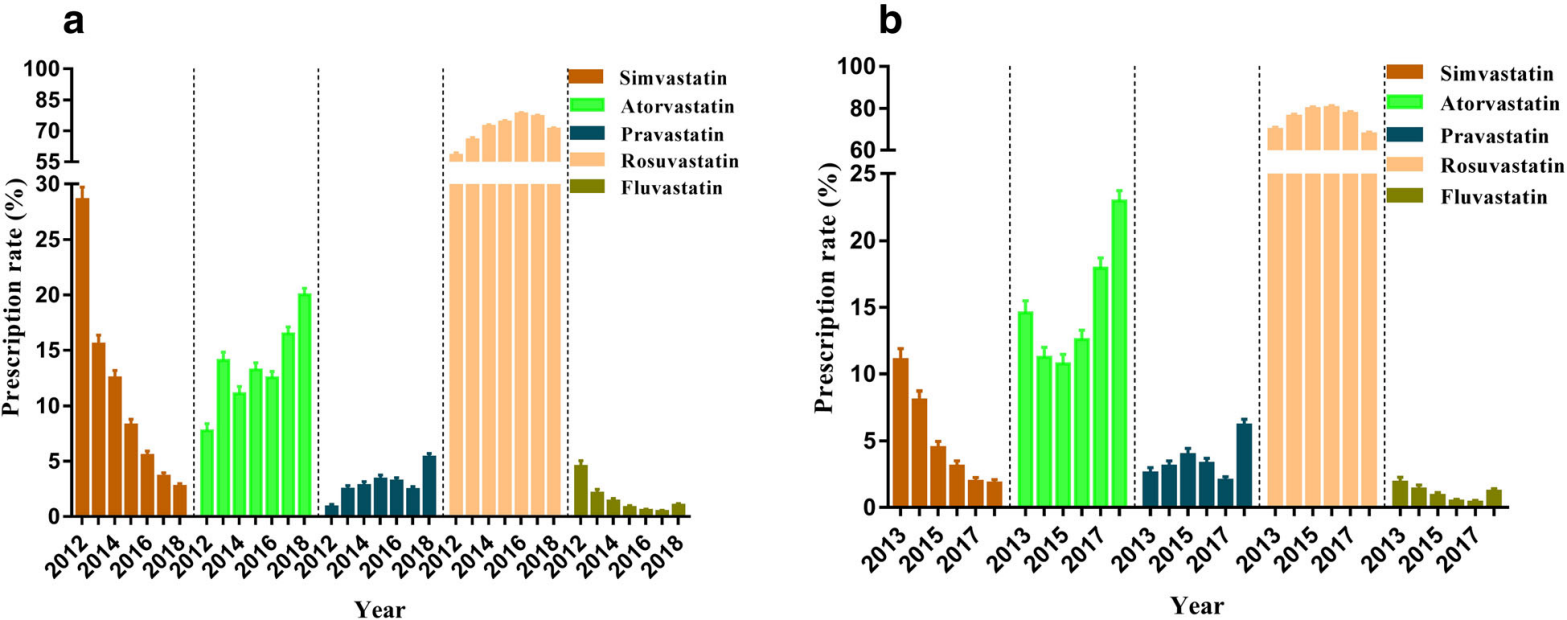
Prescription rate of statins among total statin users (**a**) and new users (**b**) from the hospital information system of Jinshan Hospital, Shanghai between 2012 and 2018. The error bars represent the upper and lower bounds of the 95% confidence intervals

The total prescription rate trend in the statin use between 2012 and 2018 was shown in Fig. 1. Rosuvastatin had an increase in its share of statin users from 2012 and 2016, from 58.23% in 2012 (95% CI: 57.02–59.44%) to 78.22% in 2016 (95% CI: 77.48–78.96%), *p* < 0.001. The prescription rate declined slightly from 2017 to 2018 (70.86, 95% CI: 70.17–71.55%). The prescribing rate of simvastatin decreased more than 14 times from 28.60% (95% CI: 27.49–29.71%) in 2012 to 2.74% (95% CI: 2.49–2.99%) in 2018, *p* < 0.001. However, the prescription rate of atorvastatin in 2012–2018 showed an overall upward trend, from 7.75% (95% CI: 7.09–8.41%) in 2012 to 20.00% (95% CI: 19.39–20.61%) in 2018, *p* < 0.001. The prescribing rate of fluvastatin was relatively small and showed a downward trend. The prescribing rate of pravastatin in 2018 was 1.02% (95% CI: 0.87–1.07%). During the study period, the rate of pravastatin increased slightly, from 0.88% (95% CI: 0.65–1.10%) in 2012 to 5.37% (95% CI: 5.03–5.71%) in 2018, *p* < 0.001.

We identified 47,924 new statin users between 2013 and 2018. The highest prescription rate of the annual use of statins in new users was rosuvastatin, with an increasing trend from 2013 to 2016 (69.87, 95% CI: 68.68–71.06% in 2013; 80.54, 95% CI: 79.68–81.40% in 2016), and slightly decreased in 2017 and 2018, *p* < 0.001 (Fig. 1). There was a significant downward trend in new statin users of simvastatin, and the prescription rate decreased from 11.07% (95% CI: 10.26 to 11.89%) in 2013 to 1.84% (95% CI: 1.59–2.09%) in 2018, roughly 6 times (*p* < 0.001). The prescription rate of atorvastatin showed an upward trend, from 14.56% (95% CI: 13.65–15.48%) in 2013 to 22.95% (95% CI: 22.17–23.73%) in 2018. The prescription rate of fluvastatin was relatively low and showed a downward trend in new statin users.

### Statin prescription prevalence

**Fig. 2:**
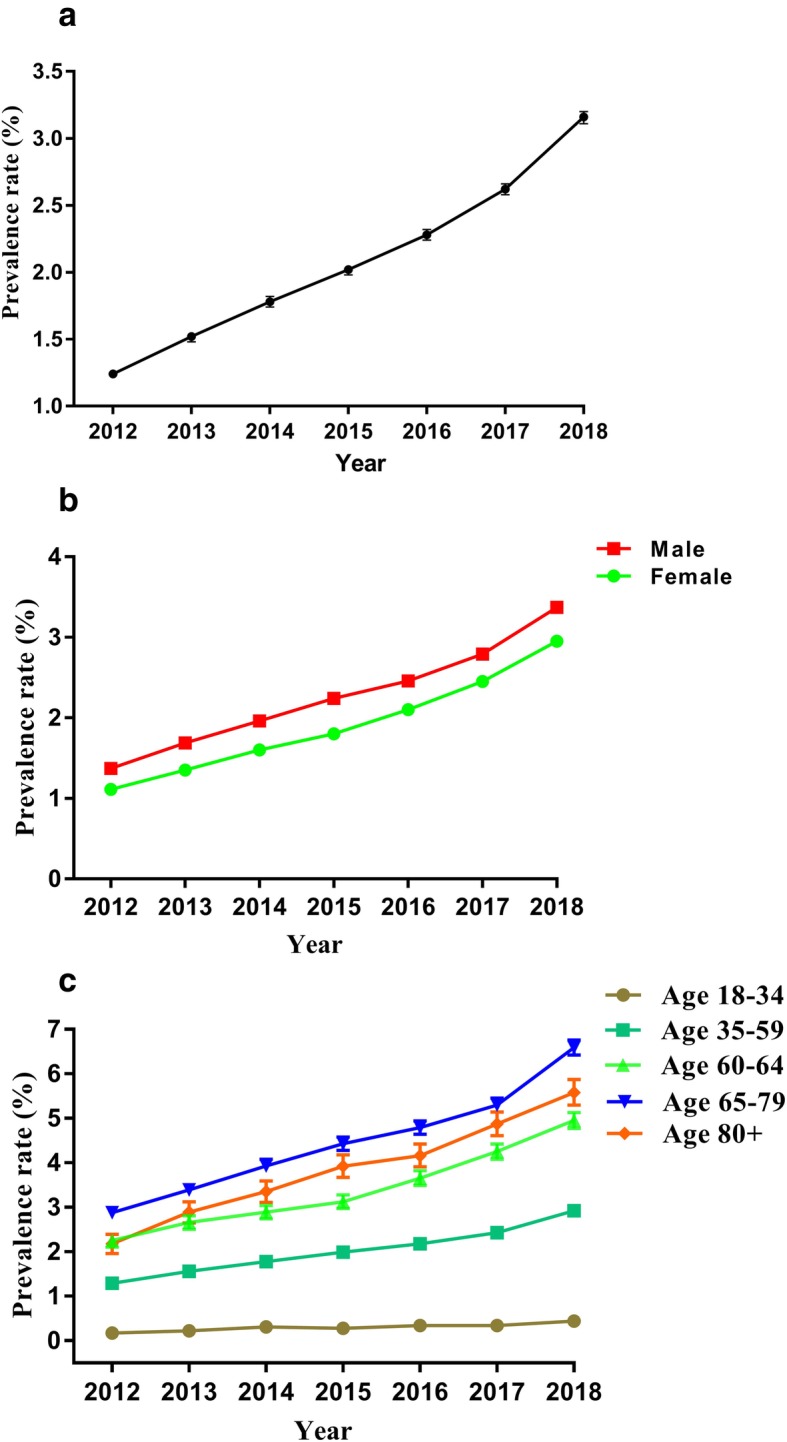
The statin prescription prevalence rates from the hospital information system of Jinshan Hospital, Shanghai (2012–2018). The error bars represent the upper and lower bounds of the 95% confidence intervals. **a** Overall prevalence rate for the total patients; **b** prevalence rate stratified according to gender; **c** prevalence rate stratified according to age

Statin prescription prevalence has increased consistently from 2012 to 2018 (test for trend in proportions, *p* < 0.001), which showed nearly a threefold in prevalence rate (1.24, 95% CI: 1.21-1.27%, in 2012 versus 3.16, 95% CI: 3.11–3.20%, in 2018) as shown in Fig. 2a.

Gender- and age-stratified analysis showed that males tended to have a higher prevalence of statin than women each year (Fig. 2b). For the 65–79 age group, they had the highest statin prescription prevalence in every year of the study, and increased from 2012 (2.88, 95% CI: 2.75–3.01%) to 2018 (6.59, 95% CI: 6.42–6.76%) (Fig. 2c). A general increase in the prescription rate was observed for other age groups between 2012 and 2018 except for the 18–34 age group.

### Statin initiation rates

**Fig. 3:**
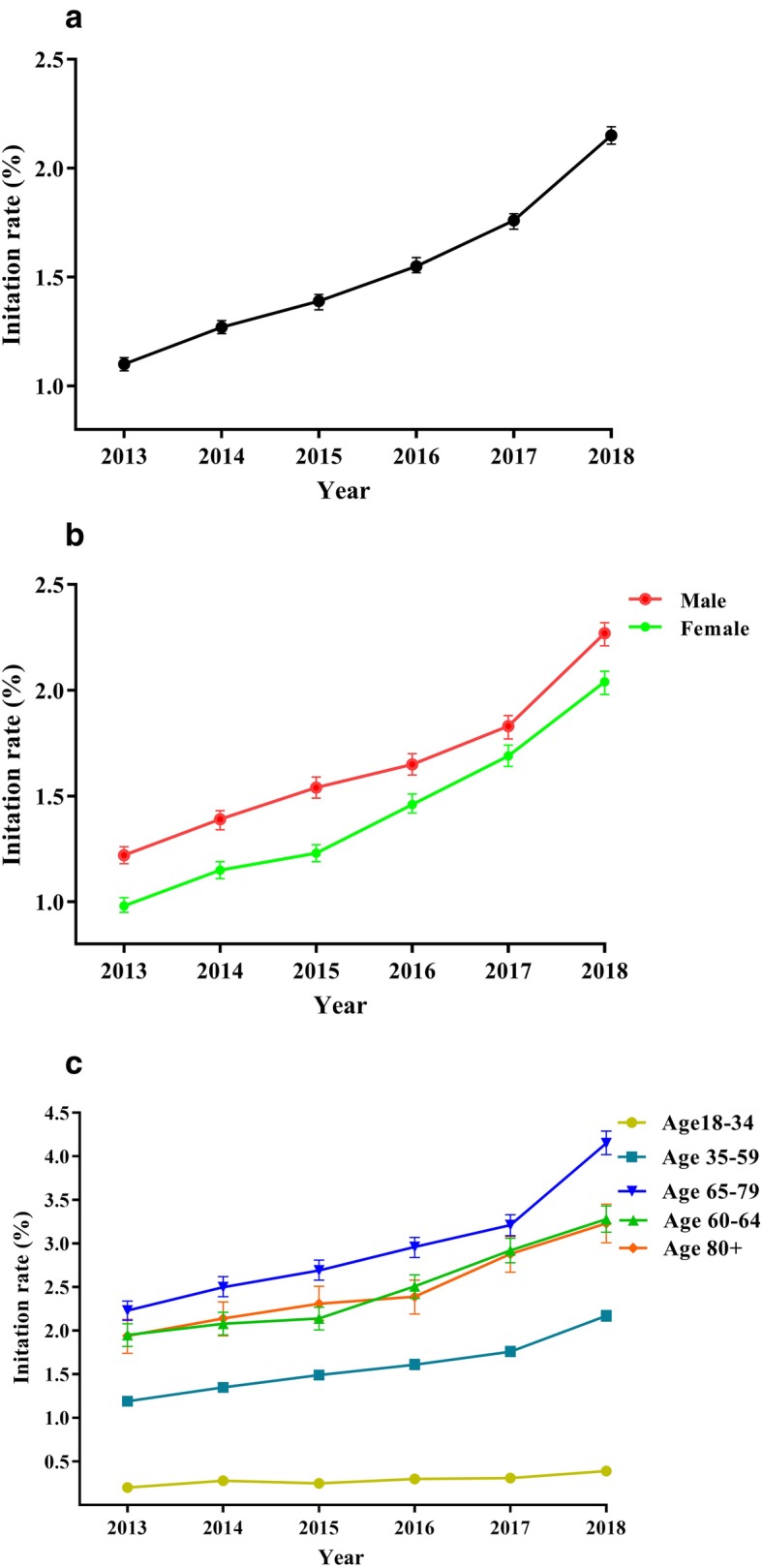
The new statin user’s initiation rates from the hospital information system of Jinshan Hospital, Shanghai (2013–2018). The error bars represent the upper and lower bounds of the 95% confidence intervals. **a** Overall initiation rate for the new users; **b** initiation rate stratified according to gender; **c** initiation rate stratified according to age

Based on 47,924 new statin users, statin initiation rate consistently increased from 1.10% (95% CI: 1.07–1.13%) in 2013 to 2.15% (95% CI: 2.11–2.19%) in 2018 (Fig. 3a). The average age at therapy initiation increased from 59.12 (±13.31) to 60.63 (±12.87) years between 2013 and 2017 and changed steadily in 2018 (60.53 ± 12.89). Women were on average 3.78 years older than men at treatment initiation (Table 2). The average age of new users is lower than that of the general population, and there was a significant statistical difference, *p* < 0.001. Among statin initiators, males have a higher initiation rate than females (Fig. 3b). When stratified according to age (Fig. 3c), there was a consistent initiation rate for new users in all age groups. There were evident rapid increases in initiation rates for 65–79 age group, which increased from 2.23% (95% CI: 2.12–2.34%) to 4.15% (95% CI: 4.02–4.29%). The second higher initiation rates were among aged 60–64 and aged above 80.

**Table 2:** The average of new statin users between 2013 and 2018 from the hospital information system of Jinshan Hospital, Shanghai

Year	Number of new users(%)	Average age (mean ± SD)	Male average age (mean ± SD)	Female average age (mean ± SD)
2013	5699 (72.67%)	59.12 ± 13.31	57.23 ± 13.55	61.43 ± 12.64
2014	6567 (71.24%)	59.10 ± 13.60	57.15 ± 14.11	61.43 ± 12.57
2015	7179 (68.70%)	59.58 ± 13.39	57.49 ± 13.84	62.16 ± 12.35
2016	8084 (68.14%)	59.95 ± 12.95	58.38 ± 13.41	61.69 ± 12.18
2017	9176 (67.01%)	60.63 ± 12.87	59.18 ± 13.60	62.19 ± 11.85
2018	11,216 (68.05%)	60.53 ± 12.89	58.96 ± 13.49	62.22 ± 11.99

### Statin dose intensity analysis

**Fig. 4:**
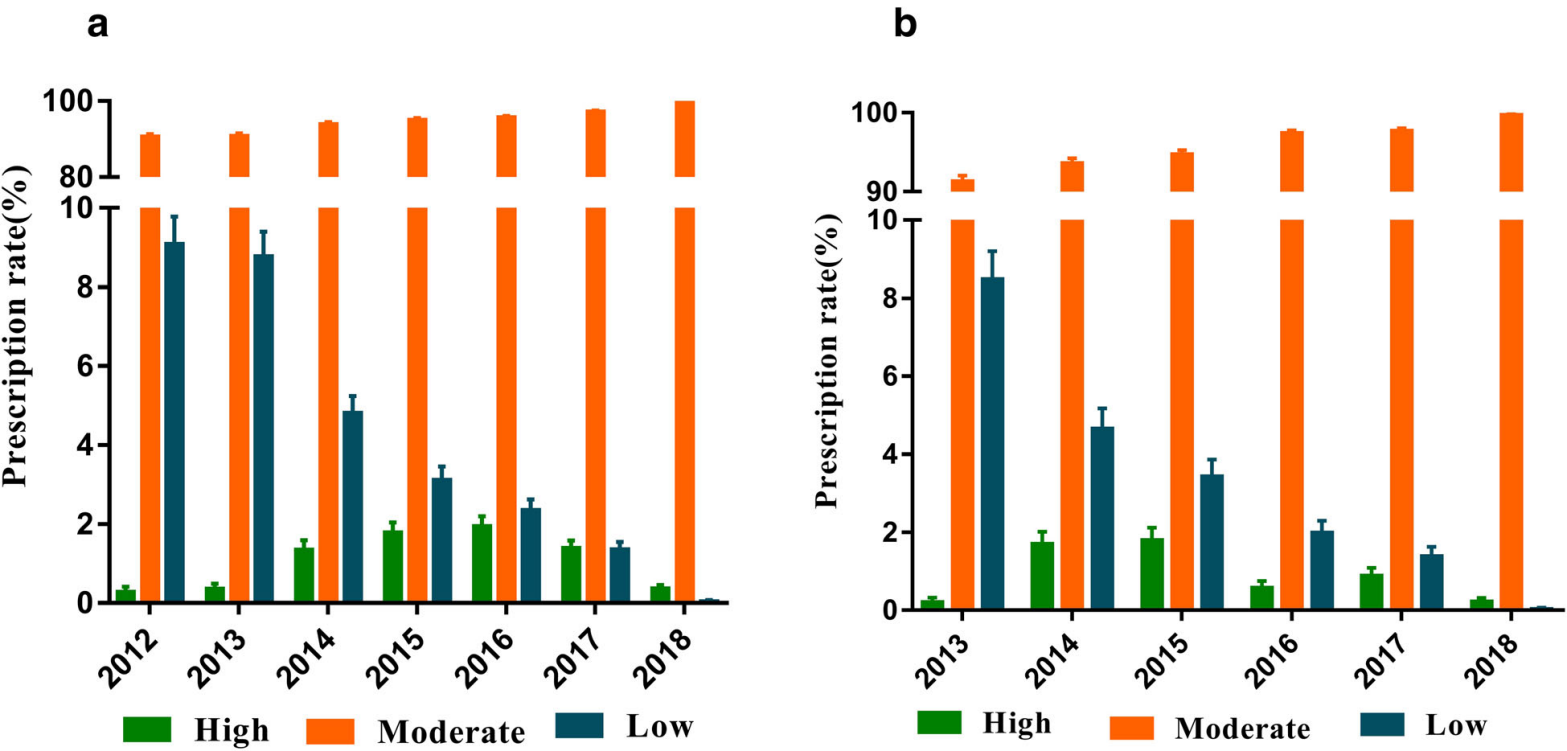
Prescription rate of statins by intensity between 2012 and 2018 in Jinshan Hospital, Shanghai. The error bars represent the upper and lower bounds of the 95% confidence intervals

Most of the prescriptions had a moderate dose of statins. Thus, the prescription rate of moderate-intensity statins gradually increased from 90.64% (95% CI: 89.93–91.35%) in 2012 to 99.59% (95% CI: 99.50–99.69%) in 2018. The prescription rate of low-intensity statins decreased from 11.13% (95% CI: 10.35–11.90%) in 2012 to 0.04% in 2018 (95% CI: 0.01–0.07%). The prescription rate of high-intensity in 2016 rose by up to 1.95% (95% CI: 1.70–2.20%), while decreased gradually and reached to 0.37% in 2018 (95% CI: 0.28–0.46%) (Fig. 4a). The new statin users were mainly prescribed with moderate-intensity therapy. The prescription rate increased gradually from 2012 to 2018. The low-intensity statin prescription rates continued to drop. The prescription rate of high-intensity statins first increased and then decreased (Fig. 4b).

### Primary and secondary prevention of cardiovascular disease

**Fig. 5:**
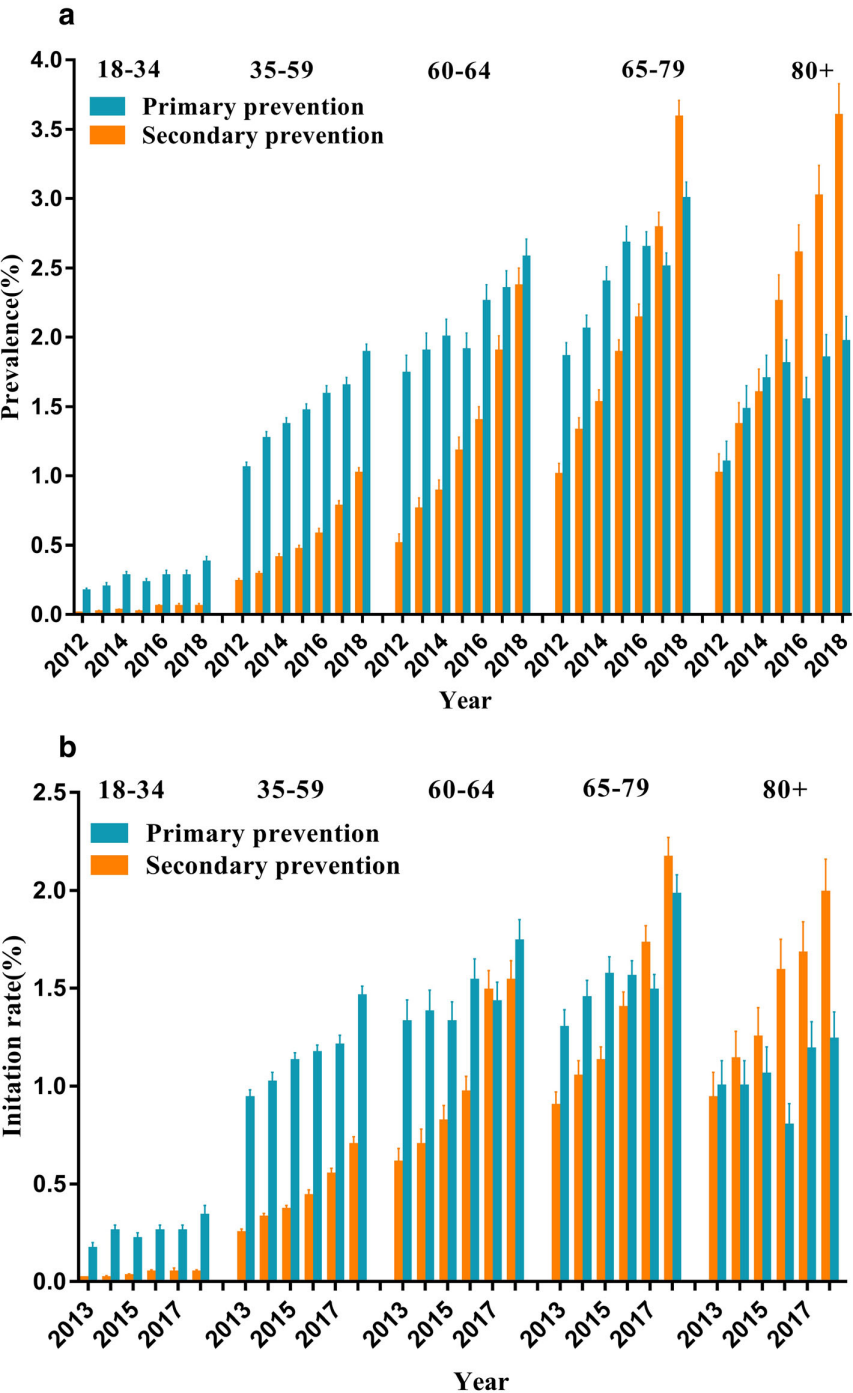
The estimated age-specific prevalence rate (**a**) for total statin users and imitation rate (**b**) for new satin users from 2013 to 2018 in Jinshan Hospital, Shanghai, stratified by cardiovascular prevention status and age group. The error bars represent the upper and lower bounds of the 95% confidence intervals

A total of 32,147 (62.93%) were taking statins for primary prevention, approximately 1.7 times of the number for secondary prevention. There was a higher percentage of use of statins among patients with hypertension, hyperlipidemia, and cerebrovascular disease (Table 3). Primary prevention occurred mainly in the age group below 60 when stratified by age. Both of the prescription rates of primary and secondary prevention increased among the 60–64 age group, but the rate of secondary prevention increased more rapidly. In 2018, the prescription rate of primary prevention is basically on par with secondary prevention, which was 2.37% (95% CI: 2.24–2.50%) and 2.58% (95% CI: 2.45–2.71%) respectively. The prescription rate of secondary prevention increased from 1.01% (95% CI: 0.94–1.09%) in 2012 to 3.59% (95% CI: 3.46–3.71%) in 2018 in the 65–79 age group (Fig. 5a). The prescription rate of primary prevention was similar to that in secondary prevention in the age group above 80 in 2012, however, the prescription rate of secondary prevention increased rapidly in 2018 (3.60, 95% CI: 3.37–3.83%). The prescription rate for new statin user prevention had a parallel trend with the total patient (Fig. 5b).

**Table 3:** The prevention status characteristics of statin users and prescription in Jinshan Hospital, Shanghai

Cardiovascular prevention status	Value(%)
Primary prevention	32,147 (62.93)
Secondary prevention	18,936 (37.07)
**Medical history**	
Cerebrovascular disease	10,095 (21.68)
Coronary artery disease	5461 (10.69)
Peripheral artery disease	2622 (5.13)
Percutaneous coronary intervention	471 (0.92)
Hypertension	14,796 (28.96)
Diabetes	5709 (11.18)
Hyperlipidemia	12,394 (24.26)
Renal disease	2042 (4.00)

**Fig. 6:**
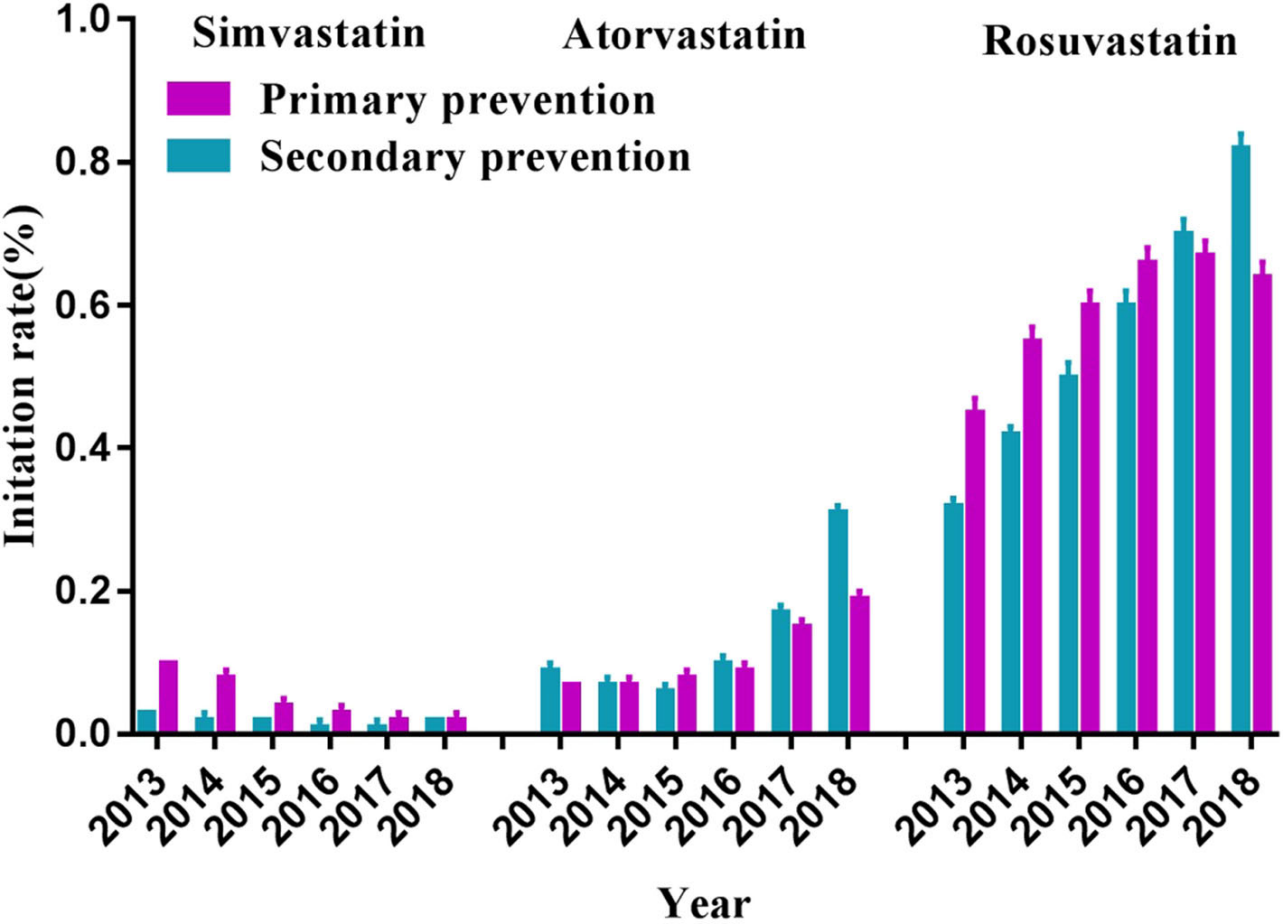
The initiation rates of new statin users stratified by simvastatin, atorvastatin and rosuvastatin from 2013 to 2018 in Jinshan Hospital, Shanghai. The error bars represent the upper and lower bounds of the 95% confidence intervals

The three most prescribed statin drugs were simvastatin, atorvastatin and rosuvastatin (Fig. 6). The initiation rate of simvastatin for primary prevention was higher than that for secondary prevention between 2013 and 2018. The initiation rate of atorvastatin for secondary prevention was slightly higher than that for primary prevention between 2013 and 2017. However, the initiation rate of atorvastatin for secondary prevention (0.31, 95% CI: 0.29–0.32%) was nearly two folds than that for primary prevention (0.19, 95% CI: 0.17–0.20%) in 2018. The initiation rate of rosuvastatin for primary prevention was higher than that for secondary prevention between 2013 and 2016, but vice versa since 2017.

## Discussion

This work is the most recent and comprehensive study on detailed statin utilization among patients using a large amount of important historical data of our hospital records. The results enable us to have detained understanding of statin prescription prevalence, therapy initiation, dose intensity, and utilization in the preventative intervention. We found that the statin prescription prevalence and initiation rate generally increased from 2012 to 2018, with most patients receiving moderate-intensity statins. Statins for primary prevention were more than that for secondary prevention. Patients with a medical history of hypertension, hyperlipidemia, and cerebrovascular events were more likely to be prescribed with statins for preventative intervention.

Statin use increased substantially in the last decade. Our finding is in line with previous studies in the United Kingdom (UK) [11], Taiwan [13], Belgium [21] and Hong Kong [14]. We speculate that the main reason may be as follow. Firstly, in recent years, the incidence of CVD continues to rise and has become the leading cause of mortality (above 40%) in China [1, 22]. Meanwhile, accumulating evidence has emerged to show the benefits of statins in the prevention of CVD, which facilitate the widespread use of statins. Secondly, the role of statins for secondary CVD prevention has been well established in several large randomized clinical trials. There has been a tendency in guidelines to offer benefits to a broad range of patients for primary prevention of CVD during the past decade [23]. However, the overall prevalence of statin use is lower from 2012 (1.24, 95% CI: 1.21–1.27%) to 2018 (3.16, 95% CI: 3.11–3.20%) in our study when compared with other studies. For instance, Joseph et al. reported the statin prevalence increased from 2004 (1.82, 95% CI: 1.78–1.86%) to 2015 (8.68, 95% CI: 8.60–8.75%) in Hong Kong, which is still lower than the prescription prevalence in the UK in 2007 (calculated as 96.53 per 1000 person-years) [11], and even lower than the 2003–2004 (11, 95% CI: 9.6–12%) and 2011–2012 (17, 95% CI: 15–19%) prevalence estimate from the US reported by Kantor et al. [24]. So, although the statin prescription prevalence was nearly doubled during the study period, the overall statins prescription rate still could not catch up that in developed countries and regions.

Besides, our study showed that the statin prescription initiation rate increased steadily and doubled in 2018. Similar patterns were observed in other studies. For instance, in Hong Kong, statin initiation rates consistently rose from 0.44% in 2004 to 1.23% in 2013 [25]. Statin therapy initiation rates in the UK increased sharply from 1995 to 2006 [11]. Another study, which was conducted in Taiwan, the initiation rate has grown from 0.6% in 2002 to 1.8% in 2011 [13]. Nevertheless, declined trends in initiation rates were observed both in Hong Kong in 2014 and 2015 and the UK in 2006. However, we did not notice such a peak in statin therapy initiation in our study, likely because of the gap in the use of statins, lack of awareness, concerns about statin adverse effects, the burden of medical cost and physician-related factors. For example, in a study investigated the change trends of statins prescription at discharge among patients with ASCVD in one of the top-ranked China hospitals, the authors found that over 40 % of patients with ASCVD were not prescribed statins when they were discharged from hospital [26]. Although, the past several years have witnessed increasing trends of statins prescription rates. Given both the low statin prescription prevalence and initiation rate, these shreds of evidence reflect the overall statin utilization rates are sub-optimal in real-world practice in China.

In the present study, we also observed a shift in statin drug choices. The most commonly used statin was rosuvastatin in our study period (2012–2018). This finding contrasts with other study results, for example, simvastatin was consistently the most prescribed statin in Hong Kong between 2004 and 2015 [14]. Atorvastatin had the highest prescription rates among new statin users throughout the study period (2002–2011) in Taiwan [13]. Interestingly, atorvastatin, simvastatin, and rosuvastatin were the most commonly prescribed statins in Asian countries and Norway [27]. Rosuvastatin had rapid annual growth in the proportion of statin users since it entered the global market in the 2000s. Prescription rates of pravastatin and fluvastatin remained relatively low. However, in recent years, we found policymakers have played a critical role in statin choice. Some European countries planned to promote prescribing of generically available statins to contain expenditure on health care. For instance, the Netherlands chose simvastatin as the reference drug to reduce costs [6]. An endorsed recommendation has also promoted a switch from atorvastatin 10 or 20 mg/day to simvastatin 40 mg/day or pravastatin 40 mg/day in the UK [28]. Furthermore, Reimbursement policies have compelled physicians to alter their statin prescription in Finland [29], Italy [30], Norway [31], Germany [32] and Austria [33]. In clinical practice, Heintjes et al. suggested the choice of statin should be based on baseline cholesterol levels to meet the reduction in it and to adapt if failed, especially in high-risk patients [25]. Therefore, as to how to choose, except for objective factors, many other factors should be taken into account, including patients’ disease conditions, cost, adverse effects, the efficacy and dose intensity of statin [3, 34–38].

Although the 2013 ACC/AHA recommend high-intensity statins for adults with ASCVD [39] and prescribing rate and initiation of high-intensity statin increased in the US after release [10, 40, 41], use of high-intensity statins remained low (under 2.1%) in Taiwan [13]. Also, there was only a little increase in the prescription prevalence of high-intensity statins from 2004 (0.05, 95% CI: 0.05–0.06%) to 2015 (0.42, 95% CI: 0.40–0.44%) [14] in Hong Kong. In our study, the peak prescription rate of high-intensity was 1.95% (95% CI: 1.70–2.20%) in 2015 and decreased gradually since then. In contrast, there was a consistent annual increase in the prescription rate of moderate-intensity statins. Also, Zhang et al. indicated that moderate-intensity statins could result in meaningful reductions in cardiovascular events [42]. More importantly, the 2016 Chinese guideline emphasized that most patients in China do not need and cannot tolerate the high-intensity high-dose statin treatment recommended by ACC/AHA, and it is clearly proposed that the low to moderate-intensity statin is more suitable for Chinese. Therefore, although some pharmacokinetic studies have demonstrated that rosuvastatin of multiple doses (5, 10, and 20 mg) was generally well tolerated in healthy Chinese volunteers [43]. However, in clinical practice, high-intensity statins are prescribed to a few patients.

There is increasing evidence supporting statin use for primary and secondary prevention of CVD [7, 11, 25]. In the recent past, our study has a witnessed significant rise in statin therapy in primary and secondary prevention. Three-fifths (62.93%) of the patients received statin therapy in primary prevention during the study period. However, the clinical benefits of statin use for primary and secondary prevention remain controversial, particularly in the elder population. A pharmacoepidemiological study in Italy revealed that although most of the patients received statin therapy in primary prevention, whereas the benefits of statins are documented mostly for patients in secondary prevention [25]. Saudi Arabia has witnessed a significant rise in CVD-related deaths in spite of the widespread use of statins in primary prevention. They also pointed out that the 2013 ACC/AHA guidelines may have overemphasized the statin therapy but without considering lipid targets which would lead to the inclusion of a large population for primary prevention with statins. Furthermore, this would cause overtreatment and potentially increase the incidences of statin-associated side effects and intolerance [8]. In addition, a statin prescription prevalence study reflects that the elderly population does not necessarily get the benefit from statin treatment for primary prevention of CVD [14]. Hence, the 2016 Chinese guideline still targets lipid levels at different stages of disease activity before recommending statins to draw guidance on the primary prevention of CVD. These findings strengthen the need to direct physicians and patients to the rational drug use of statins in the preventative intervention.

This work is the most recent and comprehensive study on statin utilization among patients using a large database of third-tier hospital. However, there are several limitations to this study. Our sample does not include data from the community hospitals and private healthcare system although the number of patients is estimated to be relatively small. Further, we would miss some samples in our estimates who may buy statins over the counter after their first visit to the hospital. A clear strength of this study is a continuous observational study with a large sample size and which reflects the local population and allows us to make an informed and comprehensive assessment of prescription prevalence, therapy initiation, dose intensity, and utilization in the preventative intervention.

## Conclusion

In conclusion, prescription prevalence and initiation generally increased from 2012 to 2018, with most patients receiving moderate-intensity statins. In recent years, males appear to have been prescribed statins at higher rates than females. However, the overall statin prescription rate in patients was lower in China when compared with other studies. The uptake of statins for both the primary and secondary prevention of CVD has increased greatly over time, in particular, in the elder group. The choice of statin should be based on patients’ actual conditions, the efficacy of statin, adverse effects and consideration to the cost performance. A coordinated effort among the patient, clinical pharmacist, stakeholders, and health system is still needed to improve statin utilization.

## Data Availability

The data that support the findings of this study are available from the corresponding author upon reasonable request.

## Abbreviations

CVD: Cardiovascular disease
TC: Total cholesterol
CHD: Coronary heart disease
ACC/AHA: American College of Cardiology /American Heart Association
ESC/EAS: European Society of Cardiology /the European atherosclerosis society
CVA: Cerebrovascular accident
LDL: Low-density lipoprotein
HIS: Hospital information system
CCEP: China cholesterol education program
ICD: International classification of diseases
